# RETRACTION: Analysis of Curative Effect of Insulin External Application on Burn Wounds of Diabetic Patients With Different Depths

**DOI:** 10.1111/iwj.70558

**Published:** 2025-04-18

## Abstract

**RETRACTION:** YangM. , LiC. , XueX. , WeiW. , XingL. , FengJ. , and ZhangQ. , “Analysis of Curative Effect of Insulin External Application on Burn Wounds of Diabetic Patients With Different Depths,” International Wound Journal 20, no. 5 (2023): 1393–1401, 10.1111/iwj.13987.36336969 PMC10088841

The above article, published online on 06 November 2022, in Wiley Online Library (http://onlinelibrary.wiley.com/), has been retracted by agreement between the journal Editor in Chief, Professor Keith Harding; and John Wiley & Sons Ltd. Following an investigation by the publisher, all parties have concluded that this article was accepted solely on the basis of a compromised peer review process. The editors have therefore decided to retract the article. The authors did not respond to our notice regarding the retraction.

**RETRACTION:** M. Yang , C. Li , X. Xue , W. Wei , L. Xing , J. Feng , and Q. Zhang , “Analysis of Curative Effect of Insulin External Application on Burn Wounds of Diabetic Patients With Different Depths,” International Wound Journal 20, no. 5 (2023): 1393–1401, 10.1111/iwj.13987.36336969 PMC10088841

The above article, published online on 06 November 2022, in Wiley Online Library (http://onlinelibrary.wiley.com/), has been retracted by agreement between the journal Editor in Chief, Professor Keith Harding; and John Wiley & Sons Ltd. Following an investigation by the publisher, all parties have concluded that this article was accepted solely on the basis of a compromised peer review process. The editors have therefore decided to retract the article. The authors did not respond to our notice regarding the retraction.

